# Application of CRISPR/Cas for Diagnosis and Management of Viral Diseases of Banana

**DOI:** 10.3389/fmicb.2020.609784

**Published:** 2021-01-27

**Authors:** Leena Tripathi, Valentine Otang Ntui, Jaindra Nath Tripathi, P. Lava Kumar

**Affiliations:** ^1^International Institute of Tropical Agriculture, Nairobi, Kenya; ^2^International Institute of Tropical Agriculture, Ibadan, Nigeria

**Keywords:** banana, viral diseases, BBTV, BSV, genome editing, CRISPR/Cas, diagnosis, disease-resistance

## Abstract

Viral diseases are significant biotic constraints for banana (*Musa* spp.) production as they affect the yield and limit the international movement of germplasm. Among all the viruses known to infect banana, the banana bunchy top virus and banana streak viruses are widespread and economically damaging. The use of virus-resistant bananas is the most cost-effective option to minimize the negative impacts of viral-diseases on banana production. CRISPR/Cas-based genome editing is emerging as the most powerful tool for developing virus-resistant crop varieties in several crops, including the banana. The availability of a vigorous genetic transformation and regeneration system and a well-annotated whole-genome sequence of banana makes it a compelling candidate for genome editing. A robust CRISPR/Cas9-based genome editing of the banana has recently been established, which can be applied in developing disease-resistant varieties. Recently, the CRISPR system was exploited to detect target gene sequences using Cas9, Cas12, Cas13, and Cas14 enzymes, thereby unveiling the use of this technology for virus diagnosis. This article presents a synopsis of recent advancements and perspectives on the application of CRISPR/Cas-based genome editing for diagnosing and developing resistance against banana viruses and challenges in genome-editing of banana.

## Introduction

Plant viruses are obligate intracellular pathogens, which utilize the host plant’s molecular machinery to replicate. They cause many economically important plant diseases and are responsible for losses in crop yield and quality worldwide. Several viruses affect banana production worldwide because of their effects on yield, quality, and limitations to the international germplasm exchange (Tripathi et al., 2016). Among these, banana bunchy top virus (BBTV, genus *Babuvirus*) and banana streak virus (BSV, genus *Badnavirus*) are economically important viruses threatening banana production (Kumar et al., 2015). These viruses reduce crop yield and productivity, posing a severe threat to food and nutrition security in banana-growing regions.

Banana and plantain (*Musa* spp., hereafter referred to as banana), is one of the chief staple food crops in 136 countries in tropics and sub-tropics, with an annual production of 155 million tons on 11 million hectares of land, and feeding millions of people (FAOSTAT, 2018). One-third of its global production is from Africa, with East Africa being the largest banana-growing and consuming region. Numerous types of banana, such as dessert, cooking, roasting, and brewing types are grown in different areas of the world and provide food for millions of people. Bananas are cultivated predominantly by smallholder farmers for home consumption and local or regional markets; only approximately 16% of production enters international markets (FAOSTAT, 2018). It is valuable food security and cash crop with huge potential to provide raw material to the budding agro-industry. It is cultivated in diverse environments and produces fruits throughout the year in favorable weather conditions.

The *Musa* spp. has four genomes corresponding to the genetic constitutions belonging to the four wild *Eumusa* species, *Musa acuminata* (AA genome), *Musa balbisiana* (BB genome), *Musa schizocarpa* (SS genome), and *Australimusa* species (TT genome; Davey et al., 2013). All cultivated bananas are generally seedless, parthenocarpic, and vegetatively propagated triploid (AAA, AAB, or ABB genome) hybrids between subspecies of *M. acuminata* (AA genome), or between *M. acuminata* and *M. balbisiana* (BB genome; McKey et al., 2010). Some cultivated bananas can have diploid or tetraploid genomes, including hybrids within or between the two *Musa* species. Hundreds of cultivars of bananas are grown and consumed worldwide. Still, large-scale farmers mainly grow the Cavendish subgroup (AAA genome) of dessert bananas for commercialization in local and international markets (Tripathi et al., 2020). Other dessert banana varieties such as Gros Michel (AAA genome), Sukali Ndiizi (commonly known as apple banana, AAB genome), Mysore (AAB genome), Silk (AAB genome), and Pome (AAB genome) are also grown at a small scale. Besides, cooking types such as the East African Highland Banana (EAHB, AAA genome) and bluggoe (ABB genome), the roasting type plantain (AAB genome), and the brewing type such as Pisang Awak (ABB genome) are also grown mainly in Africa. Plantain is mostly grown in Central and West Africa, and EAHB is cultivated in East Africa.

Banana is vegetatively propagated using suckers or *in vitro* plantlets and grown almost as perennial plantations (Kumar et al., 2015). As a vegetatively propagated crop, their production is affected due to the build−up of certain pests and pathogens, especially viruses, between successive plantings via infected planting material. Viruses of banana are challenging to control because of vegetative propagation, and many viruses are transmitted by insect vectors, contributing to further spread within the fields. Antiviral compounds are not available to cure banana plants infected with viruses. The control measures can contain the spread of viruses and prevent reinfection.

The diagnosis of a virus is the first step in the management of a viral disease. An efficient diagnostic and quarantine system is required to prevent the spread of viruses (Kumar et al., 2015). Viral infection of banana can be managed through phytosanitation, such as using virus-free planting material and strict regulation on the movement of infected planting materials. An alternative, cost-effective strategy for controlling banana viruses is to develop host plant resistance. Although conventional breeding has been used to create viral resistance in several crops, no success has been achieved in banana due to the unavailability of any known virus-resistant germplasm (Kumar et al., 2015). Developing virus-resistant varieties of banana using conventional breeding is challenging due to the low genetic variability in *Musa* germplasm, polyploidy, lengthy production cycle, and sterility of the majority of cultivars (Dale et al., 2017). Therefore, there is a critical need to delve into new breeding technologies such as transgenic and genome-editing to develop resistance against banana viruses. A few advances have been reported demonstrating enhanced resistance against BBTV using RNAi-mediated transgenic approaches (Shekhawat et al., 2012; Elayabalan et al., 2017). However, the commercialization of transgenic crops faces hurdles due to complicated regulatory approval processes. Genome-editing can fast-track breeding by making efficient and precise changes in the plant genome to develop new traits such as viral disease-resistance. A CRISPR/Cas9-based genome editing of banana has recently been established targeting the knockout of the *phytoene desaturase* (*PDS*) as a marker gene (Ntui et al., 2020). The highly efficient genome-editing tool developed using the different groups of banana has paved the path to develop disease-resistant varieties by knocking out single or multiple genes (Kaur et al., 2018; Naim et al., 2018; Ntui et al., 2020). Here, we present an overview of recent progress and perspectives to explore the application of CRISPR/Cas methods to diagnose and manage banana viruses.

## Major Viral Diseases of Banana

**FIGURE 1:**
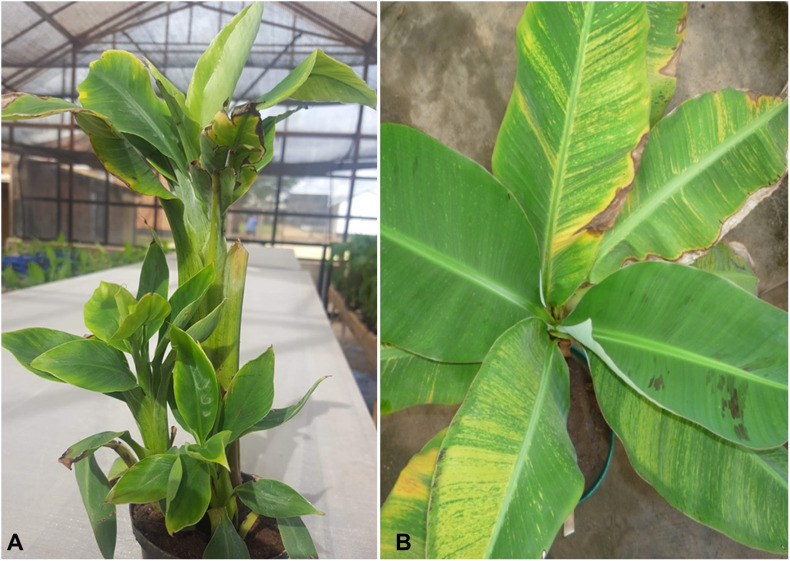
Symptoms of viral diseases in infected banana plants. **(A)** Banana bunchy top virus (BBTV) infected plant showing stunting and bunchy leaves, **(B)** Banana streak virus (BSV) infected plant showing yellow streaks.

There are about 20 viruses infecting banana globally, out of which four viruses, (BBTV, genus *Babuvirus*, family *Nanoviridae*), (BSV, genus *Badnavirus*, family *Caulimoviridae*), banana bract mosaic virus (BBrMV, genus *Potyvirus*, family *Potyviridae*) and cucumber mosaic virus (CMV, genus *Cucumovirus*, family *Bromoviridae*) are the most significant (Tripathi et al., 2016; Figure 1). Among them, BSV, BBrMV, and CMV are known to occur in all banana producing countries, whereas BBTV spread is limited to a few countries. Of all the viruses, BBTV and BSV are major threats to banana production.

**FIGURE 2:**
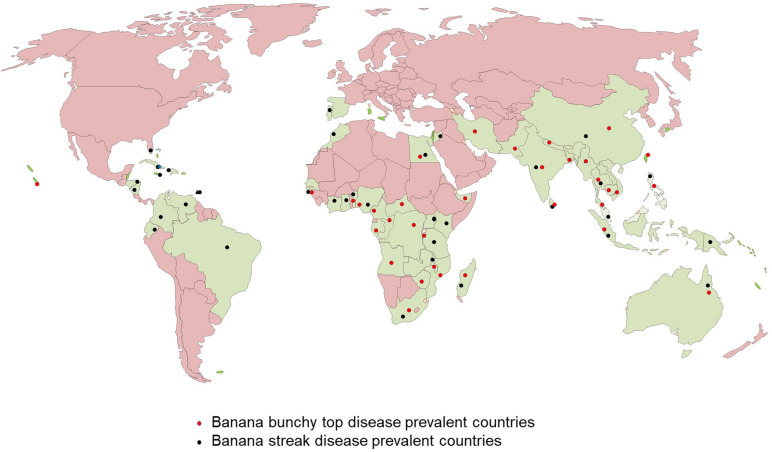
World map showing the distribution of Banana bunchy top viru*s* (BBTV) and Banana streak virus (BSV).

Banana bunchy top disease (BBTD), caused by BBTV, is the most important viral disease of banana responsible for the significant adverse economic impact on banana production. BBTV is transmitted by the banana aphid (*Pentalonia nigronervosa*) and infected planting materials. BBTD was first noticed in Fiji in the 1880s and is currently present in more than 36 countries in Africa, Asia, Oceania, and South Pacific (Kumar et al., 2015; Figure 2). In Africa, BBTD is present in 17 countries, and adjacent banana-producing countries are at a high risk of being affected (Kumar et al., 2011; Adegbola et al., 2013; Jooste et al., 2016). In the last decade, BBTV has spread to at least six countries in Africa, including Benin, Cameroon, Mozambique, Nigeria, South Africa, and Zambia. In 2018, an incidence of BBTV was reported in Togo, but early detection and eradication prevented disease establishment (IITA News, 2019). This indicates of a continuous spread of the disease in banana-producing regions, causing decreased crop production. Fruit production in infected plants reduces by 70 to 100% within one season, and plantations cannot be recovered from infections. Most cultivars of *Musa* spp. are susceptible to BBTV and few tolerant cultivars identified have limited potential for adoption in diverse production systems (Ngatat et al., 2017). The infected plants are stunted with bunchy and narrow leaves with brittle, and yellow edges (Figure 1A). Severely infected plants do not produce fruits; even if fruits are produced, they are deformed.

**FIGURE 3:**
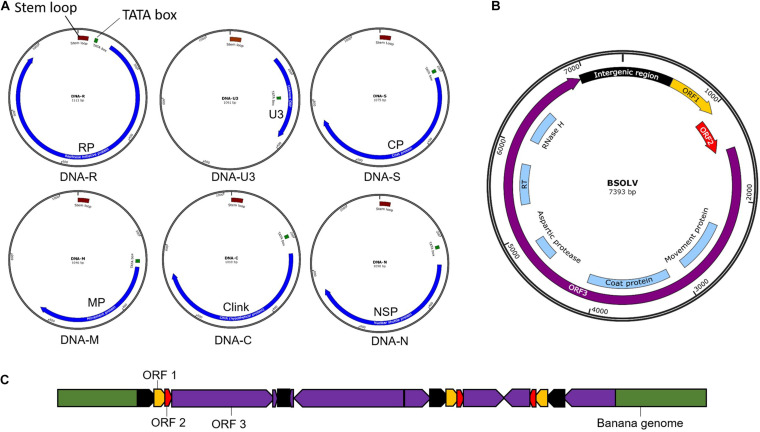
Genomic maps of banana viruses. **(A)** Banana bunchy top virus (BBTV), **(B)** Banana streak virus (BSV), and **(C)** Endogenous Banana streak virus (eBSV) integrated into the plant genome.

Banana bunchy top virus is a single-stranded DNA (ssDNA) virus with a multipartite genome comprising of six circular components (Figure 3) with an approximate size of 1.1 kb each (Harding et al., 1993; Burns et al., 1995). The six components, named DNA-R, -U3, -S, -M, -C, and -N (previously known as DNA 1-6), are encapsulated within separate virions, each about 18–20 nm in diameter (Harding et al., 1993). All the six components have a common genome organization comprising of a major common region (CR-M), stem-loop common region (CR-SL), potential TATA box 3′ of the stem-loop, at least one open reading frame (ORF) for a major gene in the virion sense and polyadenylation signals associated with each gene (Burns et al., 1995; Figure 3A). DNA-R encodes a replication initiation protein (Rep) responsible for initiating viral DNA replication, DNA-S encodes the coat protein (CP), DNA-M encodes the movement protein (MP), DNA-C encodes the cell cycle link protein (Clink), DNA-N encodes the nuclear shuttle protein (NSP), while the function of DNA-U3 is unknown (Burns et al., 1995; Wanitchakorn et al., 1997, 2000). Two broad groups of BBTV isolates have been identified based on nucleotide sequence differences between their genome components, referred to as the “South Pacific” group having isolates from Africa, Australia, South Asia, South-Pacific, while the “Asian” group comprises isolates from East Asia (China, Indonesia, Japan, Philippines, Taiwan, Thailand, and Vietnam; Karan et al., 1994; Kumar et al., 2011).

Banana streak virus is a pararetrovirus infecting banana, infection of which will result in chlorotic and necrotic streaks on leaves and pseudostem (Figure 1B). The diseased plants may be stunted with distorted fruits and smaller bunches. BSV was first reported in 1958 in West Africa and subsequently detected in all the banana-growing countries (Fargette et al., 2006; Figure 2). BSV was differentiated based on the genome sequence diversity into several species, of which the four most significant species are: *Banana streak Obino l’Ewai virus*, *Banana streak Mysore virus*, *Banana streak Imové virus*, and *Banana streak Goldfinger virus*, all of which are known to present commonly in banana plantations (Geering et al., 2005).

Banana streak virus is a bacilliform double-stranded DNA (dsDNA) virus with a monopartite genome of 7–8 Kb long with three ORFs (Figure 3B). ORF 1 encodes a small protein associated with virions (Cheng et al., 1996). OFR 2 encodes a protein (∼14 KDa) involved with virion assembly (Cheng et al., 1996). ORF 3 encodes a 208 kDa polyprotein comprising of a coat protein, MP, reverse transcriptase, aspartic protease, and ribonuclease H functions (Harper and Hull, 1998). The polyprotein is cleaved post-translationally by the aspartic protease into functional proteins.

Banana streak virus occurs in episomal and endogenous forms. The BSV genome sequence integrated into the host’s plant B genome is known as endogenous BSV (eBSV; Harper et al., 1999; Chabannes et al., 2013; Figure 3C), whereas the virus genome in the replicative form in the cell are known as episomal form. Multiple copies of eBSV sequences are integrated as direct and inverted tandem repeats at a single locus in the host B genome (Chabannes et al., 2013). The integrated form of eBSV remains dormant and develop no symptom. Under stress conditions, such as those experienced during environmental stress, micro-propagation, and interspecific crossing, eBSV becomes a functional episomal producing infectious viral particles, leading to disease symptoms in the banana plants with integrated eBSV (Cot̂e et al., 2010; Tripathi et al., 2019). The natural field transmission of BSV is through mealybugs or the use of infected planting material. However, the epidemics happen due to the activation of eBSV, not through the natural transmission.

Banana streak virus was not considered a severe threat to banana production until early 2,000. Several outbreaks of the disease reported in promising breeding lines and interspecific *Musa* hybrids as micropropagation and hybridization through conventional breeding triggered its activation (Dallot et al., 2001; Lheureux et al., 2003). Currently, BSV is considered a significant constraint in banana breeding programs, particularly for plantain (AAB genome), an important staple food in Africa. The use of the diploid progenitor *M. balbisiana* or its derivants carrying a B genome is restricted as parents for introgression of desirable agronomic traits (Duroy et al., 2016). It also limits the germplasm movement of genotypes with the B genome worldwide due to the potential activation of eBSV into the episomal infectious form of the virus. Control of BSV is difficult due to genomic integration and clonal propagation.

Cucumber mosaic virus is a positive-sense RNA virus with a tripartite genome infecting many plant species. The genome of CMV consists of three genomic RNAs (1, 2, and 3), which are necessary for systemic infections in plants (Palukaitis et al., 1992). RNA 1 and 2 are components of the CMV replicase and encode the 1a and 2a proteins, respectively. RNA 3 encodes two proteins, viral MP and the viral coat protein, expressed from subgenomic RNA 4. The MP protein facilitates the movement of CMV RNA from cell-to-cell. CMV has been found in banana-growing areas worldwide, causing chlorosis, mosaic, and heart rot (Niblette et al., 1994). CMV infection does not significantly impact banana production as BBTV or BSV; however, the infections may be severe under certain circumstances. It has been reported that CMV infection of banana has caused severe diseases in Morocco (Bouhida and Lockhart, 1990) and 100% yield losses of banana in China (Li, 1995).

## CRISPR/Cas-Based Genome Editing of Banana

Genome editing technologies facilitated by various sequence/site-directed nucleases (SDN), such as zinc-finger nucleases, meganucleases, transcription activator-like effector nucleases (TALENs), and clustered regularly interspaced short palindromic repeats/CRISPR-associated protein (CRISPR/Cas), have emerged as powerful tools for crop improvement and functional genomics. CRISPR/Cas has rapidly become the most popular genome-editing approach because of its simplicity, efficiency, versatility, specificity, and multiplexing (Scheben et al., 2017; Ntui et al., 2020; Tripathi et al., 2020).

The CRISPR-Cas system is based on the adaptive immune system of *Streptococcus pyogenes* that eliminates invasion of foreign plasmid or viral DNA. The CRISPR/Cas editing system consists of two main components: gRNA (guide RNA) and the Cas nuclease. The Cas protein exhibits nuclease activity, recognizes target DNA by gRNA-DNA pairing between the 5′ leading sequence of gRNA. It also recognizes the PAM (Protospacer adjacent motif) sequence and starts editing upstream of the sequence (Schiml and Puchta, 2016). The PAM is a three-nucleotide sequence (NGG or NAG) serving as a recognition segment for Cas to start editing upstream. The gRNA contains a scaffold and a user-defined spacer sequence (approx. 20 nt) for genomic sequence targeting. It directs the Cas to induce precise double-stranded breaks at a target site.

The natural DNA repair mechanism of the host recognizes the breaks and repairs it using the homology-directed repair (HDR) or non-homologous end joining (NHEJ) to produce the desired mutation (Weinthal and Gürel, 2016). The genome-editing takes advantage of the targeted break and the host’s natural repair mechanisms to introduce the precise, targeted changes or modifications. These modifications can be a small deletion, substitution, or the addition of nucleotides. Based on the type of repair, the editing can be SDN1, SDN2, or SDN3 (Podevin et al., 2013). SDN1 relies on the spontaneous repair of the double-stranded break by NHEJ. As NHEJ is an error-prone repair, it can lead to random mutations in the host genome, causing gene silencing, gene knock-out, or alteration in the gene function. SDN2 repairs the cleavage through HDR using a repair template complementary to the break site and containing one or few nucleotide changes and copied into the host’s genome during the repair mechanism resulting in a mutation of the target gene. SDN3 also repairs the double-stranded break via HDR using the repair template; however, in this case, the repair template is more prolonged, which might be of allelic, additional, or foreign gene, leading to the targeted insertion of the genetic material.

The types of genome-editing need to be distinguished due to potential discrepancies in the regulatory approaches. SDN1 and SDN2 are similar to mutations obtained through chemical mutagenesis, irradiation, or spontaneous natural mutations and do not lead to the insertion of foreign DNA (Schmidt et al., 2020). Any foreign DNA integrated into the plants during the genome-editing process is segregated out by crossing, especially in the seed crops. The final SDN1 and SDN2 type genome-edited products contain the desired mutations, but no foreign DNA is integrated and not considered genetically modified organisms (GMO). Segregating out the foreign gene integrated into the vegetatively propagated crop such as banana is challenging. SDN1 and SDN2 types of products with no foreign gene insertion in such crops can be obtained using the DNA-free genome-editing method, such as direct delivery of preassembled complexes of purified Cas9 protein-gRNA ribonucleoproteins (RNP) or by transient expression of the CRISPR/Cas construct. The SDN1 and SDN2 type of genome-edited products with no foreign DNA integration in the plant genome is not regulated as GMO in several countries such as Argentina, Australia, Brazil, Canada, Chile, Colombia, Japan, Israel, and the United States (Schmidt et al., 2020; Tripathi et al., 2020). However, the SDN3 type of product is subjected to regulatory controls as GMO, if the insert constitutes a foreign gene (Schmidt et al., 2020).

The CRISPR/Cas system has been extensively used for the genetic improvement of many crops (Scheben et al., 2017; Tripathi et al., 2019). Genome-edited products with improved traits can enhance yield potential by reducing the losses due to biotic and abiotic stresses. The availability of a well-annotated whole-genome reference sequence of the banana genome^1^, advancement in bioinformatics tools, and robust genetic transformation protocols (Tripathi et al., 2015) make CRISPR/Cas a suitable technology to develop disease-resistant banana.

Genome-editing in banana was first demonstrated in the cultivar “Rasthali” (AAB genome), targeting the PDS as a marker gene (Kaur et al., 2018). In this report, the authors have used a single gRNA and created mutations in the PDS gene leading to an albino phenotype. But with a relatively low mutation efficiency of 59%. In the same year, Naim et al. (2018) reported the mutation of the PDS gene in “Cavendish Williams” (AAA genome) with higher editing efficiency of 100% using polycistronic gRNAs. Similarly, Ntui et al. (2020) demonstrated high mutation efficiency of 100% using multiple gRNAs targeting the PDS gene in banana cultivar “Sukali Ndiizi” (AAB genome) and plantain cultivar “Gonja Manjaya” (AAB genome). The PDS gene is generally used as a visual-marker gene for establishing genome-editing systems in plants. The PDS gene encodes a key enzyme in the carotenoid biosynthesis pathway and converts phytoene into carotenoid precursors phytofluene and ζ-carotene. The disruption of its function leads to albino phenotype, which is a visible indicator. However, the knockout of PDS negatively affects plant growth. Alternatively, Zorrilla-Fontanesi et al. (2020) used *RP43/CHAOS39*, a gene encoding the chloroplast signal recognition particle (cpSRP) machinery, as a visual marker to optimize the genome-editing protocol for banana. The *CHAOS39* edited banana plants showed pale-green phenotypes with normal *in vitro* growth. Nevertheless, the researchers need to be careful in using *cpSRP43/CHAOS39* as a visual marker as the pale green phenotype can be achieved with other factors such as nutrient deficiency and improper light.

Our laboratory has established a robust genome editing platform for banana and plantain by using the multiplexed CRISPR/Cas9 system. This platform is now routinely used for the generation of genome-edited banana and plantain for disease resistance. Application of CRISPR/Cas9-based genome-editing system for banana was demonstrated by inactivating the endogenous eBSV sequence integrated into the B genome of plantain using multiple gRNAs (Tripathi et al., 2019). Recently, CRISPR/Cas9 technology was applied to create mutations in the *M. acuminata gibberellin* 20ox2 (MaGA20ox2) gene to develop semi-dwarf plants of the banana cultivar “Gros Michel” by disrupting gibberellin production (Shao et al., 2020).

## Application of CRISPR/Cas for Diagnosis of Banana Viral Diseases

Sensitive and reliable diagnostic tools for detecting BBTV and BSV are crucial for surveillance programs, clean planting material production, and phenotyping. Many serological and nucleic acid-based methods have been established to detect these two viruses (Kumar et al., 2015). Polymerase chain reaction (PCR)-based methods are widely used for the detection of BBTV and BSV. However, immunocapture PCR or rolling circle amplification methods are used for distinguishing episomal and endogenous forms of BSV. The PCR-based methods offer reliable detection of the two banana infecting viruses, but they require sophisticated equipment and laboratory facilities, limiting the adoption of these methods. The ability of CRISPR/Cas precise targeting of nucleotide sequences has been harnessed to develop highly sensitive and rapid isothermal diagnostic tools to detect viruses, bacteria, and cancer diagnosis (Chertow, 2018; Khambhati et al., 2019). Similar to genome editing, a gRNA aids in the specific recognition of a target nucleic acid sequence and activates enzymatic cleavage of the target nucleic acid by the Cas enzyme, which is then detected using a chromogenic or fluorometry detection system.

The CRISPR/Cas9-based tools were first used to detect the Zika virus after isothermal amplification of the target RNA (Pardee et al., 2016). In this method, the viral RNA was amplified by reverse transcription (RT)-PCR or RT isothermal amplification methods. The amplified product was detected using the CRISPR/Cas9 cleaving of the amplified DNA and the results were visualized by colorimetric toehold using RNA switch sensors. An improved method for the same virus using CRISPR/Cas9 triggered isothermal exponential amplification reaction (CAS-EXPAR) offered detection of viral genome at attomolar (aM) sensitivity and single-base specificity capable of differentiating African and American strains by colorimetric detection using SYBR Green florescence signal (Huang et al., 2018). The discovery of RNA-guided, RNA-targeting CRISPR effector Cas13a, and subsequently identified Cas12a, Cas13b, and Cas14a facilitated CRISPR-Cas12a, Cas13a, Cas13b, and Cas14-based nucleic methods for the detection of several human-infecting viruses and bacterial pathogens (Gootenberg et al., 2017, 2018). Of these, Cas13 types are suitable for direct detection of homologous RNA targets using RNA guide, and the Cas12 and Cas14 types are appropriate for the detection of single-stranded and dsDNA-targets, respectively, as these enzymes direct RNA guides to homologous DNA targets. A diagnostic procedure named the “Specific High Sensitivity Enzymatic Reporter UnLOCKing (SHERLOCK)” system was developed to detect the target sequence by isothermal amplification of target molecule with Recombinase Polymerase Amplification (RPA)/Reverse Transcriptase (RT)-RPA or Loop-mediated Isothermal Amplification (LAMP)/RT-LAMP (Gootenberg et al., 2017). The target amplicons are then subjected to *in vitro* T7 transcription, followed by the detection of RNA molecules generated by Cas13-guided reporter assay. The final products are detected by chromogenic detection on a later-flow device or with a fluorometer when fluorescent-labeled probes are used (Gootenberg et al., 2018). As a further improvement, the HUDSON (heating unextracted diagnostic samples to obliterate nucleases) method was standardized for the direct detection of viruses in the bodily fluid samples without nucleic acid extraction step for sensitive, rapid, and instrument-free detection of target viral nucleic acid molecule by pairing with the SHERLOCK (Myhrvold et al., 2018). This method was shown to be sensitive for detection of virus species, strains, and clinically relevant mutations directly, without nucleic acid extraction to speed up the virus detection Myhrvold et al., 2018). The Cas12a and Cas12b enzymes were also used for developing CRISPR/Cas based diagnostics. The Cas12a recognizes T rich PAM sequence for targeted cleavage of dsDNA targets using a method termed DETECTR (DNA endonuclease targeted CRISPR *trans*-reporter; Chen et al., 2018). In this, target RNA or DNA molecule is amplified by PCR/RT-PCR or isothermal amplification methods (RPA/RT-RPA, LAMP/RT-LAMP). The amplified dsDNA products are detected using the sg-RNA-Cas12a complex, which triggers the degradation of the ssDNA fluorophore-quencher reporter probe. The results are detected by colorimetric or fluorometry detection (Chen et al., 2018). The Cas14a enzyme function similar to that of the Cas12 system, except that it detects ssDNA (Harrington et al., 2018). Most of the CRISPR-Cas diagnostics involve pre-amplification of the target molecule by PCR or RT-PCR, or isothermal amplification methods such as RPA or RT-RPA, LAMP, or RT-LAMP depending on the type of target virus genome. The target molecules are detected using the signals generated from fluorophore-quencher-based reporter RNA molecule or by separating reactions on lateral flow devices or SYBR Green fluorescence detection system (Wang et al., 2019). For BBTV, an Exo-RPA isothermal detection system targeting the BBTV-R DNA segment has already been developed and standardized for the virus detection directly in the plant tissue without the need for DNA extraction (IITA News, 2019). The amplified products are detected using the FAM-labeled Exo-RPA probes in a fluorometer (Khambhati et al., 2019). Efforts are ongoing to convert the BBTV Exo-RPA assay into a HUDSON-SHERLOCK detection system for low-cost, rapid, and sensitive detection of the virus both under laboratory and field conditions. Simultaneously, existing PCR-based methods for BSV detection are being converted to CRISPR/Cas diagnostics to detect BSV and its variants in banana. Because of high specificity and sensitivity for detecting near single copy of the target molecules, CRISPR-based diagnostics tools have the potential to offer highly robust, rapid, and sensitive detection of banana viruses for seed health certification, surveillance, and other applications.

Recently, an efficient and rapid RT-RPA-CRISPR/Cas12a system has been developed as a one-step detection assay to diagnose plant RNA viruses (Aman et al., 2020). This diagnostic assay uses a fluorescence visualizer to detect the plant RNA viruses. It can be performed in less than 30 min at a single temperature in the field. This assay could be used quickly and efficiently to detect banana RNA viruses fast-tracking their containment strategies.

## Control of Viral Diseases Using CRISPR/Cas

The CRISPR/Cas system is becoming the method of choice to control plant viruses, either by targeting the viral factors for viral genome editing in viruses or by targeting the host plant factors responsible for the viral cycle. CRISPR/Cas-based genome editing for controlling plant viruses is reported for ssDNA viruses, dsDNA viruses, and ssRNA viruses (Baltes et al., 2015; Ali et al., 2016; Hadidi et al., 2016; Zaidi et al., 2016; Zhang et al., 2018; Gomez et al., 2019; Tripathi et al., 2019; Table 1). Although most of the application of the CRISPR/Cas to develop virus-resistance is reported for ssDNA and dsDNA viruses in plants, its application against RNA viruses is also reported (Zhao et al., 2020). The CRISPR/Cas-based resistance to plant RNA viruses is based on the editing of host plant factors influencing viral infection rather than the viral genes. The plant host factors like eukaryotic translation initiation factor (eIF) are required to maintain replication of RNA viruses. Several eIF such as eIF4E and eIF(iso)4E, have been identified as recessive resistance alleles to confer resistance against several potyviruses (Khatodia et al., 2017). The genome-edited plants with mutations in the eIF(iso)4E gene demonstrated enhanced resistance against cucumber vein yellowing virus, cassava brown streak virus, Ugandan cassava brown streak virus, papaya ringspot virus-type W, zucchini yellow mosaic virus, and turnip mosaic virus (Chandrasekaran et al., 2016; Pyott et al., 2016; Gomez et al., 2019).

**TABLE 1:** Summary of developing virus resistance in plant species using CRISPR/Cas-based genome-editing.

**Plant**	**Editing system**	**Target gene**	**Target trait**	**Outcome**	**References**
Arabidopsis	CRISPR-Cas9	*eIF4E*	Turnip mosaic virus (TuMV)	Complete resistance to TuMV	Pyott et al., 2016
Arabidopsis	CRISPR-Cas9	*CP*	Cauliflower mosaic virus (CaMV)	Resistance to CaMV	Liu et al., 2018
Arabidopsis	CRISPR-Cas9	Viral genome of CMV	Cucumber mosaic viru*s* (CMV), Tobacco mosaic virus (TMV)	Resistance to CMV and TMV	Zhang et al., 2018
Banana	CRISPR-Cas9	ORF1, ORF2, and ORF3 of BSV	Banana streak virus (BSV)	Inactivation of integrated endogenous BSV (eBSV)	Tripathi et al., 2019
Barley	CRISPR-Cas9	*CP, MP, Rep, RepA*	Wheat dwarf virus (WDV)	Resistance/tolerance to WDV	Kis et al., 2019
Cassava	CRISPR-Cas9	*nCBP-1 and nCBP-2*	Cassava brown streak virus *(CBSV)*, Ugandan cassava brown streak virus (UCBSV)	Reduction in disease symptom severity and incidence	Gomez et al., 2019
Cassava	CRISPR-Cas9	*AC2 and AC3*	African Cassava mosaic virus (ACMV)	No resistance against ACMV	Mehta et al., 2019
Cucumber	CRISPR-Cas9	*eIF4E*	Cucumber vein yellowing virus (CVYV), Zucchini yellow mosaic virus (ZYMV), Papaya ringspot virus-type W (PRSV-W)	Resistance to multiple viruses (CVYN, ZYMV, PRSV-W)	Chandrasekaran et al., 2016
Potato	CRISPR-Cas13a	*P3*, *CI*, *Ni*, *CP*	Potato virus Y (PVY)	Suppressed PVY accumulation and disease symptoms	Zhan et al., 2019
Rice	CRISPR-Cas9	*eIF4G*	Rice tungro spherical virus (RTSV)	Resistance to RTSV	Macovei et al., 2018
Rice	CRISPR-Cas13a	Three regions in viral genome	Southern rice black-streaked dwarf virus (SRBSDV)	Reduction in disease symptoms	Zhang et al., 2019
Rice	CRISPR-Cas13a	Three regions in viral genome	Rice stripe mosaic virus (RSMV)	Reduction in disease symptoms	Zhang et al., 2019
Tobacco	CRISPR-Cas9	Coding and non-coding region of TYMV	Tomato yellow leaf curl virus (TYLCV)	Significant reduction in disease symptoms	Ali et al., 2015
Tobacco	CRISPR-Cas9	*CP, Rep*	Cotton leaf curl kokhran virus (CLCuKoV)	Reduced viral activities	Ali et al., 2016
Tobacco	CRISPR-Cas9	*CP, RCRII*	Merremia mosaic virus (MeMV)	Reduced viral activities	Ali et al., 2016
Tobacco	CRISPR-Cas9	*6* regions in the viral genome	Bean yellow dwarf virus (BeYDV)	Reduction in viral accumulation	Baltes et al., 2015
Tobacco	CRISPR-Cas9	Viral genome	Beet severe curly top virus (BSCTV)	Reduction in viral accumulation	Ji et al., 2015
Tobacco	CRISPR-Cas9	*IR, CP, Rep*	Tomato yellow leaf curl virus (TYLCV)	Resistance to TYLCV	Tashkandi et al., 2018
Tobacco	CRISPR-Cas9	Viral genome of CMV	Cucumber mosaic virus (CMV), Tobacco mosaic virus (TMV)	Resistance to CMV and TMV	Zhang et al., 2018
Tobacco	CRISPR-Cas13a	*4* regions of TuMV	Turnip mosaic virus (TuMV)	Virus interference	Aman et al., 2018
Tobacco	CRISPR-Cas9	*IR, V2/V1, C1/C4*	Chili leaf curl virus (ChiLCV)	Reduction in viral accumulation	Roy et al., 2019
Tobacco	CRISPR-Cas9	*IR and CI* coding regions	Cotton leaf curl multan virus (CLCuMV)	Complete resistance to CLCuMV	Yin et al., 2019
Tobacco	CRISPR-Cas9	*Rep*,β*C1*	Cotton leaf curl virus (CLCuV)	Delayed symptom development and reduction in viral accumulation	Khan et al., 2020
Tomato	CRISPR-Cas9	*IR, CP, Rep*	Tomato yellow leaf curl virus (TYLCV)	Resistance	Tashkandi et al., 2018

Banana bunchy top virus is a multipartite ssDNA virus that replicates either by the host or virally encoded DNA polymerases through a dsDNA intermediate form during replication. In contrast, BSV is a monopartite dsDNA virus, with a DNA-RNA intermediate during replication similar to that of pararetrovirus replication. The dsDNA structure of both viruses makes them a good target for CRISPR/Cas9 mediated genome editing. Although no progress on CRISPR/Cas mediated resistance against BBTV has been reported, several investigations have documented the use of CRISPR/Cas9 to induce durable virus resistance to ssDNA viruses through gene knockout in many plant species (Yin and Qiu, 2019; Kalinina et al., 2020; Table 1). These mechanisms could be harnessed to develop resistance to BBTV in banana.

CRISPR/Cas-mediated resistance to DNA viruses was first demonstrated for geminiviruses (Ali et al., 2015; Baltes et al., 2015; Ji et al., 2015). The CRISPR/Cas9 technology was applied for developing resistance against beet severe curly top virus (BSCTV, genus *Geminivirus*) in Arabidopsis and *Nicotiana benthamiana*, targeting the replication-associated protein (Rep), coat protein (CP), and intergenic region (IR; Ji et al., 2015). The genome-edited plants demonstrated high resistance against BSCTV with up to 87% reduction in viral load. Similarly, a high level of resistance against the bean yellow dwarf virus (BeYDV, genus *Mastrevirus*) in *N. benthamiana* using CRISPR/Cas9 plasmid construct targeting the *Rep* gene delivered through *Agrobacterium*-mediated transformation was recorded (Baltes et al., 2015). The edited plants expressing CRISPR-Cas reagents showed targeted mutations within the viral genome and demonstrated reduced virus load and symptoms upon challenge with BeYDV. These studies reported novel strategies for controlling geminiviruses.

In another approach, *N. benthamiana* was engineered with resistance against the tomato yellow leaf curl virus (TYLCV, genus *Begomovirus*), a monopartite begomovirus, by transiently delivering a CRISPR/Cas9 construct targeting the viral *Rep*, *CP*, and the conserved region of IR of TYLCV using the tobacco rattle virus (TRV) vector (Ali et al., 2015). The *N. benthamiana* plants exhibited resistance to TYLCV. Further, stable transgenic edited plants were generated through *Agrobacterium*-mediated transformation using the same CRISPR/Cas9 construct. These transgenic *N. benthamiana* plants exhibited broad-spectrum resistance against the monopartite beet curly top virus (genus *Curtovirus*), the bipartite Merremia mosaic virus (MeMV, genus *Begomovirus*), and TYLCV. Later, *N. benthamiana* was engineered using a CRISPR/Cas9 system to interfere with the coding sequences of TYLCV, MeMV, and cotton leaf curl Kokhran virus (genus *Begomovirus*; Ali et al., 2016). However, this led to the emergence of a new mutated virus variant, which evaded the CRISPR/Cas9 activity, and viruses continued to replicate and spread systemically. Further, when the IR sequences were targeted, the new mutated virus variants were not detected, suggesting that targeting non-coding viral sequences may be better for controlling multiple geminiviruses simultaneously using CRISPR/Cas. Similarly, tomato plants exhibiting resistance against TYLCV were produced using the CRISPR/Cas9 system targeting the IR region (Tashkandi et al., 2018).

Recently, complete resistance against the cotton leaf curl virus (CLCuV, genus *Begomovirus*) was demonstrated in *N. benthamiana* using a CRISPR/Cas9 system by multiplexing gRNAs targeting *Rep* and IR sequences (Yin et al., 2019). Later, *N. benthamiana* plants agroinfiltrated with CRISPR/Cas9 construct having multiplex gRNAs targeting the *Rep*, and β*C1* gene of the betasatellites showed delayed disease symptoms and lower titer of CLCuV (Khan et al., 2020). Interestingly, the inactivation of the *AC2* gene encoding the transcription activator protein and the *AC3* gene encoding the replication enhancer protein of African cassava mosaic virus (ACMV, genus *Begomovirus*) failed to produce resistance against the virus in transgenic cassava (Mehta et al., 2019). The authors reported that CRISPR editing led to the formation and escape of new CRISPR-resistant ACMV variants, which were probably generated due to the post cleavage NHEJ repair.

Genome-edited barley plants with resistance to the wheat dwarf virus (genus *Mastrevirus*) were generated by multiplexing four gRNAs targeting the overlapping region of *MP* and *CP* coding sequence, *Rep/RepA* coding region at the N-terminus of the proteins, long intergenic region (LIR) region, and the genomic region encoding the C-terminus of Rep (Kis et al., 2019). Similar approaches using CRISPR/Cas systems targeting *MP, CP*, and *Rep* can be applied in banana to enhance resistance against BBTV. Improved resistance to BBTV by silencing the *CP, MP*, and *Rep* using the RNAi approach was demonstrated (Shekhawat et al., 2012; Krishna et al., 2013; Elayabalan et al., 2017). However, the transgenic banana plants only showed partial resistance to BBTV. It might probably be because RNAi does not always result in a complete knockout; therefore, genome-editing could potentially be used to simultaneously knocked out several genes. The banana plants resistant to BBTV can be developed by delivering the CRISPR/Cas9 reagents targeting either the viral genes such as *CP, MP*, and *Rep*, or the host plant factors involved in viral infection like *eIF* gene.

The CRISPR/Cas system has also been used to successfully provide resistance against other DNA viruses, besides ssDNA viruses. For example, a CRISPR/Cas9 system was used to enhance resistance against cauliflower mosaic virus (genus *Caulimovirus*), a dsDNA pararetrovirus, in *Arabidopsis* by creating targeted mutations in the coat protein (Liu et al., 2018). However, the authors also reported some mutated forms of the virus, which could escape and spread in systemically infected leaves.

CRISPR/Cas technology can also be applied to pararetroviruses such as BSV or retroviruses with dsDNA as part of their replication. Currently, the application of CRISPR/Cas9 to disrupt both episomal and integrated dsDNA viruses is reported for only one plant virus (Tripathi et al., 2019). However, it has been demonstrated for control of several human viruses, including papillomaviruses (HPV16 and HPV18), hepatitis B virus, Epstein-Barr virus (EBV), HIV-1, polyomavirus 2 (John Cunningham Virus), Herpes simplex virus-1, and other herpesviruses (White et al., 2016). CRISPR/Cas system has been used to treat HIV-1 infection by targeting both viral and host factors (Chen et al., 2018). HIV is a retrovirus integrating the viral DNA into the host DNA and reactivates, causing HIV-AIDS. CRISPR/Cas9 technology was used to inactivate the HIV-1 by knocking out proviral DNA integrated into the host cells by targeting long terminal repeat (LTR) flanking sequences or overlapping ORFs or multiple regulatory genes within the HIV-1 provirus (Wang et al., 2016; Wang et al., 2016; Ophinni et al., 2018). CRISPR/Cas9 targeting the LTR significantly suppresses the activation of HIV-1; however, viral escape was also reported (Wang et al., 2016). The probability of the virus escape could be overcome by multiplexing gRNAs targeting different ORFs (White et al., 2016). Similarly, CRISPR/Cas9 targeting multiple ORF targets (E6 and E7) was used to inactivate HPV, a dsDNA virus that gets integrated into the chromosomes of host cells (Kennedy et al., 2014; Zhen et al., 2014). Similarly, a CRISPR-SpCas9 tool was applied, targeting six different regions in the EBV genome (Wang and Quake, 2014). Subsequently, Yuen et al. (2015) multiplexed a CRISPR/Cas9 system targeting a 558 bp fragment in the promoter region of BART (*Bam*HI A rightward transcript) and a primary viral transcript encoding the viral microRNAs (miRNAs).

Similarly, a multiplex CRISPR/Cas9 system targeting all three ORFs of BSV was used to inactivate the integrated dsDNA of eBSV from the banana genome (Tripathi et al., 2019). The CRISPR/Cas9 construct with multiple gRNAs targeting the ORF1, ORF2, and aspartic protease gene of ORF3 was delivered into the host cells of plantain “Gonja Manjaya” through *Agrobacterium*-mediated genetic transformation to inactivate the virus. The regenerated genome-edited plantain with targeted mutations in the viral genome prevented proper transcription or/and translation into infectious viral proteins. Phenotyping of the potted genome-edited plants under water stress conditions in the greenhouse confirmed the inactivation of the virus as 75% of the tested plants remained asymptomatic under stress conditions; in contrast, the wildtype control plants showed BSV disease symptoms. All the asymptomatic plants have mutations in all the three ORFs. The application of CRISPR/Cas9-mediated genome editing of banana for controlling viruses is only reported for BSV.

There are reports of the emergence of new mutated virus variants in plants and human using the CRISPR-Cas9 with a single sgRNA targeting viral genes (Ali et al., 2016, Wang et al., 2016, Mehta et al., 2019). The emergence of mutated new variants of the viruses, which may be hypervirulent, can be delayed by targeting multiple viral genes for editing, using the more efficient versions of Cas9, targeting the non-coding region of virus genome, or deleting the larger portions of the viral genome (White et al., 2016; Mehta et al., 2019). The variability in the virus sequences also plays a role in circumventing resistance in the plants expressing the CRISPR machinery targeting viral genes. The CRISPR-Cas9 system should be carefully designed to engineer virus resistance to minimize chances of viral escape.

## Challenges in Genome-Editing of Banana

Banana is a polyploid heterozygous crop containing a high number of multigene families with paralogs (Cenci et al., 2014). One of the significant challenges of genome editing in a banana is to target multiple alleles and gene copies simultaneously. Sometimes knockdown or knockout of a particular gene does not result in any phenotypic change, maybe due to the dose-effect of other paralogous copies of genes. Therefore, the gRNA needs to be designed to target all the copies and alleles of the gene and screen many mutants to recover an edited line with multiallelic mutations. Multiplexed genome-editing using multiple gRNAs targeting several genes and their paralogs in a gene family can be an efficient tool for improving polyploid crops (Ansari et al., 2020).

Another major challenge is that genome editing in a banana crop is currently achieved by plasmid-based delivery through *Agrobacterium* transformation (Ntui et al., 2020). In other crops, the CRISPR reagents are delivered through a range of transformation methods, such as protoplast transfection, agroinfiltration, and stable transformation through *Agrobacterium* and microprojectile bombardment (Nadakuduti et al., 2018). However, transient delivery systems like agroinfiltration or protoplast fusion are not successful in banana. The mutants generated through stable transformation are considered GMOs due to transgene integration in the plant genome and had to go through time-consuming regulatory approvals, and this could reduce their acceptability. Since banana is a vegetatively propagated crop, segregating out of the Cas9 gene, marker gene, and *Agrobacterium*–derived DNA sequences through backcrossing is not feasible due to the sterility of the majority of the farmer-preferred cultivars, unlike seeded crops (Nadakuduti et al., 2018; Tripathi et al., 2020). To overcome these biosafety concerns, there is a need to develop transgene-free genome-edited banana plants. Recently, several efforts have been put to produce transgene-free genome-edited plants by directly delivering preassembled Cas9 protein-gRNA RNP, otherwise known as RNPs, into plant cells (Woo et al., 2015; Malnoy et al., 2016; Svitashev et al., 2016; Liang et al., 2018; Tripathi et al., 2020). Upon delivery, the RGENs-RNPs edit the target sites immediately and are rapidly degraded by endogenous proteases in cells, leaving no traces of foreign DNA elements (Kanchiswamy et al., 2015; Woo et al., 2015).

In banana, preassembled RGENs-RNPs targeting viral genes or plant host factors could be coated to gold particles and delivered to banana cell suspension cultures by particle bombardment. Alternatively, the CRISPR/Cas9 constructs targeting the viral genes or plant host factors could transiently be delivered into banana cells through microprojectile bombardment. The complete plants can be regenerated from the bombarded banana cells. The edited plants with the targeted mutations and absence of foreign gene integration should be selected based on the molecular characterization. The virus-resistant genome-edited plants generated using preassembled RGENs-RNPs or transient delivery of CRISPR/Cas9 reagents will not have any foreign gene integration and might not require GMO regulatory approval (Tripathi et al., 2020).

The major biosafety concerns with genome-edited crops are unwanted genetic changes in plants due to off-target mutations and transgene integration. The off-target effects can be minimized by improving strategies for designing the gRNA very specific to the target and RNP-based delivery as they are active for a short duration in the host cell and use of inducible CRISPR/Cas system to avoid the strong doses of CRISPR-Cas9 beyond the target cells/tissues and throughout the life span of the plant.

Another pressing issue is the lack of high throughput screening methods to identify genome-edited events. Currently, the edited plants of banana are screened using PCR and target sequencing to detect the mutation. However, these methods are expensive and time-consuming. Screening the edited plants using the high-throughput phenotyping for the desired trait, followed by target sequencing of the selected events, will be more efficient and cost-efficient.

The challenges with the differences among the countries regarding the regulation of genome-edited products cannot be ignored (Tripathi et al., 2020). The genome-editing products with no foreign gene integration are not regulated in many countries (Schmidt et al., 2020). Only the EU and New Zealand consider genome-edited products under the existing GMO biosafety (Schmidt et al., 2020).

## Conclusion

Clustered regularly interspaced short palindromic repeats/CRISPR-associated protein-based genome editing is fast revolutionizing its applications in crop improvement for desired traits such as disease resistance. BBTV and BSV are economically important viruses threatening banana production. The most sustainable way to reduce losses due to these viral diseases is the use of virus-resistant banana varieties. In the absence of any known resistant germplasm, it requires the development of durable virus-resistant varieties using modern biotechnological tools, complementing conventional breeding. Genome editing tools provide a new weapon in the arsenal against plant viruses. The availability of a robust genome-editing system and reference genome make the banana a potential candidate for developing virus-resistant varieties using CRISPR/Cas-based genome editing. So far, genome-editing is applied to banana only for control of BSV by inactivating the eBSV integrated into the host genome. CRISPR/Cas system targeting the essential genes of the virus or host plant genes involved in the susceptibility can be applied in banana to develop resistance against BBTV. Genome-edited virus-resistant banana varieties can be generated with no foreign gene integration, making them more acceptable for commercialization.

## Author Contributions

LT conceived the idea. All authors contributed to writing, reviewing, and editing the manuscript.

## Conflict of Interest

The authors declare that the research was conducted in the absence of any commercial or financial relationships that could be construed as a potential conflict of interest.

## Footnotes

1 http://banana-genome-hub.southgreen.fr
